# The Influence of the Degradation of Tetracycline by Free Radicals from Riboflavin-5′-Phosphate Photolysis on Microbial Viability

**DOI:** 10.3390/microorganisms7110500

**Published:** 2019-10-28

**Authors:** Shiuh-Tsuen Huang, Shwu-Yuan Lee, Song-Hua Wang, Chun-Yi Wu, Jeu-Ming P. Yuann, Sin He, Chien-Wei Cheng, Ji-Yuan Liang

**Affiliations:** 1 Department of Science Education and Application, National Taichung University of Education, Taichung 40306, Taiwan; hstsuen@mail.ntcu.edu.tw; 2 Department of Tourism and Leisure, Hsing-Wu University, New Taipei City 24452, Taiwan; 091007@mail.hwu.edu.tw; 3 College of Life and Health Science, Anhui Science and Technology University, Fengyang, Chuzhou 239000, China; shwang70@163.com; 4 Department of Biotechnology, Ming-Chuan University, Gui-Shan 33343, Taiwan; kevin1060755@gmail.com (C.-Y.W.); jyuann@mail.mcu.edu.tw (J.-M.P.Y.); peter100d16@gmail.com (S.H.)

**Keywords:** blue light, tetracycline, free radicals, riboflavin-5′-phosphate, photolysis

## Abstract

Tetracycline (TC) is a broad-spectrum antibiotic compound. Wastewater with TC may have an adverse effect on ecosystems. Riboflavin-5′-phosphate (FMN or flavin mononucleotide) is a non-toxic product of the phosphorylation of vitamin B_2_ and is required for the proper functioning of the humans. FMN is sensitized to ultraviolet (UV) and blue light radiation, as evidenced by the generation of reactive oxygen species (ROS). This study inspects feasible applications of blue light on FMN so as to develop a valid way of degrading TC by FMN photolysis. We used the increased rate of bacterial survival as a practical indicator of antibiotic degradation. TC in the presence of FMN solution decomposed completely after 20 W/m^2^ of blue light irradiation (TCF treatment), and the degradation of TC (D-TCF) occurred after the photolytic process. After TCF treatment, colony-forming units (CFUs) of *Escherichia coli* (*E. coli)* were determined for the D-TCF solution. The CFU of *E. coli* preservation was 93.2% of the D-TCF solution (50 μg/mL of TC in the presence of 114 μg/mL of FMN solution treated with 20 W/m^2^ of blue light irradiation at 25 °C for 1 h) cultivation. The mass spectrum of D-TCF showed diagnostic ion signals at m/z 431.0 and 414.0 Da. The molecular formula of D-TCF was C_21_H_22_N_2_O_8_, and the exact mass was 430.44 g/mol. TC degradation by FMN photolysis can significantly decrease the antimicrobial ability of TC. The results expressed here regarding the influence of FMN photolysis on TC degradation offer an environmentally sound wastewater treatment method.

## 1. Introduction

The term, “tetracyclines” (TCs), represents a group of broad-spectrum antibiotic compounds. The name tetracycline (TC) comes from its tetracyclic nucleus, which is composed of four linear attached hydrocarbon rings. A variety of functional groups bonded to the nucleus can lead to a tendency toward antibiotic behavior. Therefore, TC is a broad-spectrum antibiotic that can be used against numerous microorganisms, such as Gram-positive and -negative bacteria, protozoan parasites, and so forth. TC interferes with the binding of aminoacyl-transfer ribonucleic acid (aminoacyl-tRNA) to the bacterial ribosome and hence suppresses the synthesis of proteins within microorganisms [1]. TC is widely used in the livestock business for disease prevention, which makes it an important growth promoter of livestock [2]. However, more than 70% of the TC used by humans is excreted through the urine and feces of humans or animals into the environment, after systemic administration.

The Gram-negative bacterium, *E.* *coli,* along with coliforms, was discovered in the digestive systems of animals. *E. coli* is a pathogen indicator for drinking water and food systems. It is also an indicator of environmental systems. Waste containing TC may present a severe hazard to ecosystems [3]. Therefore, removing TC from waste has become an important issue in relation to maintaining ecosystems.

It is well established that TC is sensitive to ultraviolet (UV) light. Reactive oxygen species (ROS) are generated from TC photolysis under simulated solar irradiation [4]. TC can absorb UV light and be degraded by UV irradiation [5]. Antibiotic photoreaction under solar irradiation is considered a significant approach to degradation in natural aquatic environments [6]. TC is unstable when treated with visible light and is also sensitized to blue light irradiation in an alkaline environment. Anionic superoxide radicals (O_2_•^−^), in addition to other photolytic products of TC, are generated during the electron-transfer mechanism of TC photolysis [7]. Under blue light irradiation treatment, TC has been used in the photodynamic therapy technique by yield O_2_•^−^, which is highly reactive to bacteria inactivation [7].

ROS, for example, the hydroxyl radical (•OH) and superoxide radical (O_2_•^−^), are normally reactive and small species [8]. Riboflavin (vitamin B_2_, RF) and riboflavin-5′-phosphate (FMN) are required by humans. They are required by all flavoproteins during energy generation cycles [9]. FMN is produced from the phosphorylation of riboflavin. Both FMN and riboflavin are sensitized to light irradiation [10]. After irradiation under UV [9,10,11,12,13] and blue light [14,15], these two compounds have been shown to attain an excited state via photolysis. The photo-activated riboflavin is transformed into its excited triplet state, and ROS is simultaneously generated [10,16]. Riboflavin photolysis was used for the expansion of a secure and valid method for inactivation of pathogens in blood [14,15]. It was reported that HeLa cells treated with FMN or flavin adenine dinucleotide (FAD) under blue light irradiation were subjected to apoptosis through the concomitant ROS production for poisoning tumor cells in vitro [17]. Our previous investigation showed that the inactivation of *E. coli* and *staphylococcus aureus* can be achieved by DNA cleavages induced by O_2_•^−^ production via riboflavin or FMN photolysis [14,15]. The riboflavin photolytic reaction has been used to degrade crystal violet (CV), a triphenylmethane dye and an antibacterial agent via ROS generation, leading to a huge decrease in the antimicrobial ability of CV [18].

Heavy metal catalysts, such as titanium dioxide [19,20], strontium titanate [21], zinc oxide [22], ferric oxide [23], and ferrous or ferric iron/hydrogen peroxide [24], as well as UV irradiation have been applied to decompose TC. In addition, the structural changes of TC may alter its molecular property and, accordingly, influence the antibiotic effectiveness of a photochemical process. However, UV radiation and heavy metals are toxic. It has been reported that the antimicrobial ability of TC is decreased by approximately 22% when TC is treated in the presence of riboflavin solution under Xe or quartz-halogen lamp irradiation for 40 min [25]. Nevertheless, both FMN and riboflavin are sensitized to light irradiation [10]. The water solubility of FMN is enhanced by approximately 200 times, compared with that of riboflavin [10]. However, the levels of ROS production were shown to be higher in FMN than those in riboflavin under blue light irradiation at the same energy dosage [15]. It would be of interest to determine whether adding FMN photolysis can change the structure of TC via ROS generation, which, in turn, would result in a huge decrease in the antimicrobial ability of TC.

In our previous studies, we found that TC is sensitive to blue light irradiation, and the self-photosensitization of TC occurs by the O_2_•^−^ generated via TC photolysis [7]. TC itself has negligible effects on multidrug-resistant (MDR) *E. coli* survival. However, following blue light irradiation treatment, the degraded TC significantly inactivates MDR *E. coli.* The structural modifications of TC can therefore alter its antibiotic property [7]. It has been reported that degraded TC can produce a higher stress hazard under UV irradiation via photolytic intermediates [26]. Wastewater containing TC may damage the microbial environments of ecosystems. Thus, microbial viability can be applied as a necessary indicator to evaluate the influences caused by TC pollution. 

TC and FMN are both sensitized to blue light, and O_2_•^−^ is produced via photolytic processes. Further research may compare the ROS generation ability of TC and FMN under blue light irradiation under the same conditions. It would be of interest to investigate the structural changes and the antimicrobial ability of TC via the FMN photolytic process. Thus, if TC degraded by FMN photolysis can largely reduce the antimicrobial ability of TC, a potential and an effective protocol treatment could be obtained.

Treated with blue light irradiation, FMN can degrade TC and eliminate the antimicrobial ability of TC. This study investigated the O_2_•^−^ generation from TC and FMN irradiated with blue light. Additionally, the effects of FMN photolysis and TC self-photosensitization on TC degradation and structural changes were examined as well. A potential and an effective protocol was developed on the basis of TC degradation via FMN photolysis and treatment with blue light irradiation. The bacterial viability caused by degraded TC provides an index that indicates the impact of the technology.

## 2. Materials and Methods

### 2.1. Chemicals 

**Figure 1:**
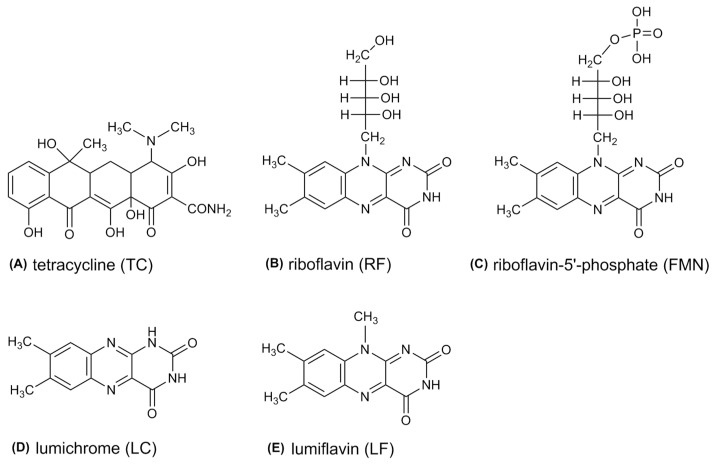
The chemical structure of (**A**) tetracycline (TC), (**B**) riboflavin (RF), (**C**) riboflavin-5′-phosphate (FMN), (**D**) lumichrome (LC), and (**E**) lumiflavin (LF).

TC contains a straight fused tetracyclic nucleus. Structures of TC, riboflavin, FMN, lumichrome (LC), and lumiflavin (LF) are shown in Figure 1. l-Methionine, FMN, potassium dihydrogen phosphate, dipotassium phosphate, and TC were acquired from Sigma-Aldrich Co. (St. Louis, MO, USA). Nitro blue tetrazolium chloride (NBT) was purchased from Bio Basic Inc. (Markham, Ontario, Canada). The ultra-pure water, used as the solvent throughout this study, was prepared by a Milli-Q system.

### 2.2. Set-Up of the Photolysis System 

**Figure 2:**
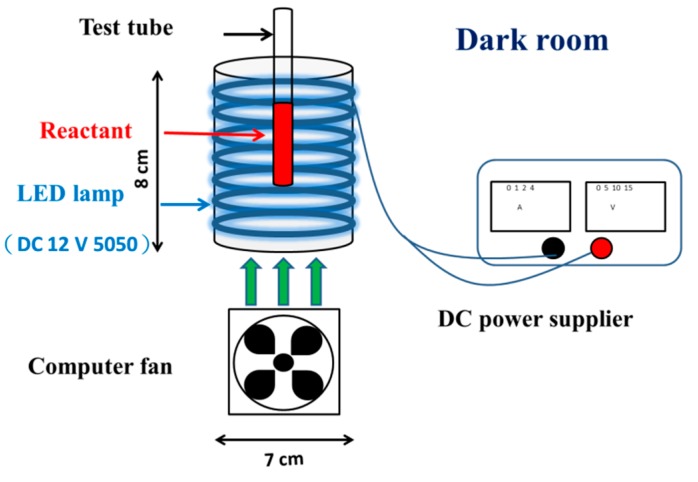
Experimental setup of the photoreaction system. LED: light-emitting diode.

The photochemical reactions were conducted in a plastic cylinder cup and set in a dark room, as described previously [7,27,28]. Six blue light-emitting diode (LED) lights (12V/5050 LED) were purchased from Vita LED Technologies Co. (Tainan, Taiwan). The LED lights were applied as the illuminator by surrounding the inner part of the cup. The emitted maximum wavelength of blue light was 462 nm, and the spectral width at half height (W_1/2_) was 23 nm, as measured in a previous study [7]. The TM-207 solar power meter made by Tenmars Electronics Co. (Taipei, Taiwan) was employed as an irradiance controller. The experimental setup of the photoreaction system is shown in Figure 2.

### 2.3. FMN or TC under Blue Light Photoreaction 

This study surveyed the blue light effects on FMN and TC degradation. The reaction solution was added to a test tube and positioned on top of the cup. The blue light effects on the photoreactions of FMN and TC were estimated by a UV/VIS spectrometer (Lambda 35, Perkin-Elmer, Waltham, MA, USA). The reaction solution was either 50 μg/mL of TC or 50 μg/mL of FMN in 0.1 M phosphate buffer solution (PBS, pH 7.8). Briefly, (i) reaction solutions of TC and FMN in the dark separately were treated as a control group, (ii) the reaction solution of either TC or FMN was maintained under blue light irradiation at 20 W/m^2^ for 0–30 min, and (iii) a different batch of the reaction solution of 50 μg/mL of TC mixed with 50 μg/mL of FMN in PBS was maintained under blue light irradiation at 20 W/m^2^ for 0–30 min at 10 ± 1 °C in a cold room. The absorbance of the reaction solutions was surveyed at 300–800 nm via UV/VIS.

### 2.4. Detection of O_2_•^−^

O_2_•^−^ can be detected by direct or indirect methods. The direct method uses a more delicate instrument, for example, electron paramagnetic resonance (EPR) spectroscopy. The indirect methods are better for examination [28]. Indirect methods, such as the reduction of nitro blue tetrazolium (NBT), have been applied to measure the O_2_•^−^ activity in riboflavin or FMN photoreactions [27,29]. NBT is reduced by the O_2_•^−^ generated from intermediates [30] and is an indirect assay used to inspect O_2_•^−^. Under blue light irradiation, this study investigated the impact of the molecular alterations of TC and FMN on O_2_•^−^ production. 

The reaction solutions were made fresh, prior to the experiments. Initially, 109.3 mg of l-methionine was mixed with 75 mL of PBS. Afterward, 25 mL of 200 μg/mL TC (or 200 μg/mL FMN) and 10 mg of NBT were added to the mixed solution, that is, the final concentrations of TC (or FMN), l-methionine, and NBT were 50 (50), 1090, and 100 mg/L, respectively. The reaction solutions were irradiated at 20 W/m^2^ by blue light for different time intervals at room temperature. The O_2_•^−^ produced from the photolytic reactions reduced NBT to yield formazan detected at 560 nm. 

### 2.5. Influences of TC and Degraded TC on Bacterial Viability 

In our previous study, 50 μg/mL of TC had insignificant efficacy on MDR *E. coli* survival at 10 and 25 °C [7]. The changed conformation of TC after blue light irradiation and self-photosensitization can therefore alter its antibiotic property, and the change configuration of TC maintained the antibacterial ability of MDR *E. coli* through photolysis [7]. In our previous study, blue light-induced photolysis with riboflavin or FMN was found to generate ROS to inactivate *E. coli* and lead to the degradation of crystal violet (CV) [18]. The cultivation of bacteria and the TC (FMN) photochemical reaction may increase, along with the reaction temperature, at 10 and 25 °C. The structural changes of TC under the FMN photolytic process and the changed structure of TC retain or reduce the antimicrobial ability at different temperatures and are therefore interesting to investigate.

The involvement of both TC and degraded TC in the antibacterial ability of *E. coli* were examined in this study. The irradiation conditions were the same as those described in Section 2.3. In brief, (i) PBS was left in the dark and served as a control, (ii) 20 or 50 μg/mL of TC in PBS was left in the dark, and (iii) 20 or 50 μg/mL of TC with 114 μg/mL of FMN in PBS was maintained under blue light irradiation at 20 W/m^2^ for 2 h (TCF treatment; energy dose, 14.4 J/cm^2^; 1 W × h = 3600 J, 20 W/m^2^ × 2 h = 144,000 J/m^2^ = 14.4 J/cm^2^). Meanwhile, the reaction settings were maintained at 10 ± 1 °C. As for the TCF treatment, experiments were performed at room temperature, that is, 25 ± 3 °C for 0.5 (energy dose, 3.6 J/cm^2^) or 1 h (energy dose, 7.2 J/cm^2^) to inspect how temperature influences *E. coli* inactivation.

The DH5α strain of *E. coli* (NCBI 668369) was developed for laboratory cloning use and cultivated in Luria Bertoni (LB) media at 37 °C overnight. After harvest, 500 μL of bacterial broth loaded put in a 1.5 mL centrifuge tube and diluted with water. Then, cultures were cultivated to reach an optical density of 0.5 at 600 nm (OD_600_), for which the bacterial density was about 4.1 × 10^7^ CFU/mL. The supernatant was removed after centrifugation for 5 min. Prior to resuspension, 1 mL of TC or degraded TC from the TCF treatment (D-TCF) was added to the pellet, whereas the PBS without TC was used as a control. The bacterial solution in the presence of TC or D-TCF was then incubated for 1 h. Then, 200 μL of bacterial solution was placed on Luria agar plates and cultured at 37 °C overnight. The *E. coli* survival rate after each treatment was inspected by the viable plate count. The inactivation rate of *E. coli* was calculated according to the reducing percentage of the viable cells, (1 − C/N) × 100%, where C and N are the numbers of CFUs for each test of either TC or degraded TC treatment (C) and no TC treatment (N), respectively. Then, the reduction rate of CFUs was expressed as the negative numerical value of the inactivation rate.

### 2.6. TC and D-TCF Analysis by LC-MS/MS 

The FMN photolytic reaction on TC degradation was analyzed using an LC-MS/MS method. The separation of TC and FMN in the solutions is described in Section 2.3. The samples were maintained under blue light irradiation at 20 W/m^2^ for 1 h. TC, FMN, and D-TCF were eluted by an Agilent Poroshell 120 EC-C18 column (2.7 µm, 4.6 mm × 150 mm, Agilent Technologies, Palo Alto, CA, USA), followed by LC-MS/MS analysis. Using an Agilent 1200 Series HPLC System, an electrospray ionization (ESI) source was connected to an Agilent 6410B triple quadrupole MS in this study.

The mobile phase was composed of 0.1% methanoic acid (A) and methanol (B). The process of separation was completed by a mobile phase, the profile of which is depicted below. The linear gradient started with 0–2 min, 95%–80% A; 2–8 min, 80% A; 8–12 min, 80%–40% A; 12–15 min, 40% A; 15–19 min, 40%–5% A; 19–24 min, 5% A; and 24–27 min, 5%–95% A. The final mobile phase was set at 95% solvent A from 27 to 30 min. The reaction solution at a volume of 10 μL was injected at a flow rate of 0.4 mL per minute. TC or D-TCF was investigated by a positive-ion mode for charged fragments. The data were obtained and inspected using the MassHunter Workstation software, version B.06.00.

### 2.7. Statistics

The results were expressed as the mean ± standard deviation (SD) of at least three individual tests. A homoscedastic two-tailed Student’s *t*-test was used for the determination of differential significance between two measurements. The difference between two groups was considered statistically significant (*p <* 0.05).

## 3. Results

### 3.1. Spectral Effects of Blue Light on FMN and TC 

**Figure 3:**
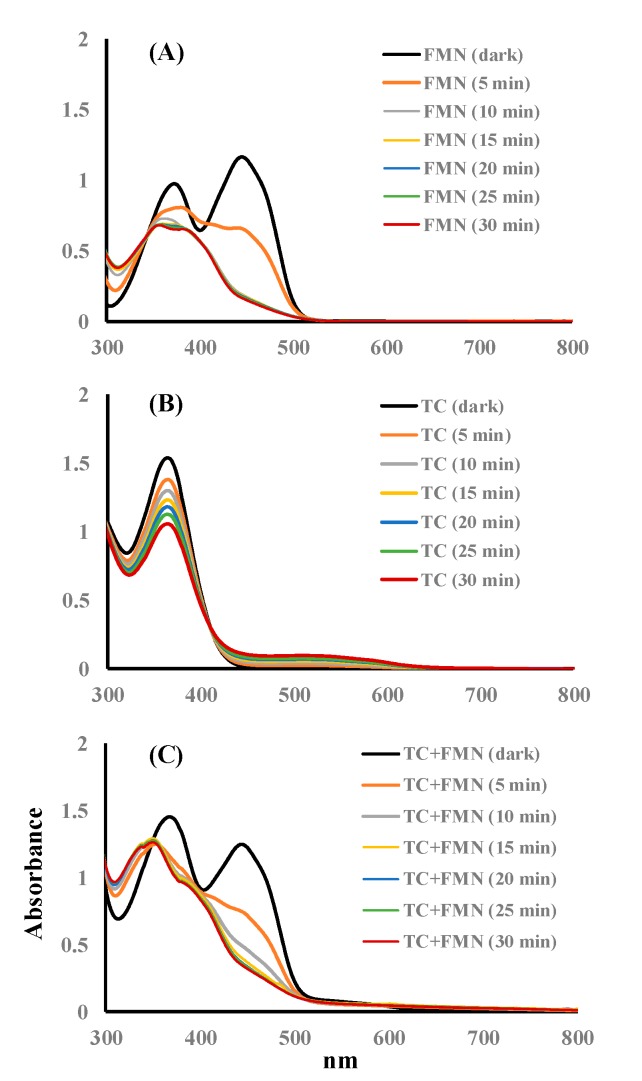
Absorption spectra after 0–30 min of blue light irradiation at 20 W/m^2^ of (**A**) 50 μg/mL of FMN, (**B**) 50 μg/mL of TC, and (**C**) 50 μg/mL of FMN in the presence of 50 μg/mL of TC. The absorbances of the reaction solutions were collected in the wavelength from 300 to 800 nm.

The spectra of the FMN and TC solutions treated with blue LED lamp for 0–30 min are shown in Figure 3. In the FMN solution, two maxima at 373 and 445 nm were observed in the absorption spectrum of the FMN dark control, and the peak at 445 nm was significantly reduced after 10 min of blue light irradiation, as shown in Figure 3A. In the TC solution, one peak at 364 nm was detected for the dark control, and the absorption at 364 nm was extensively decreased after blue light irradiation for 30 min. The spectra of the TC in the FMN solution under blue light irradiation for 0–30 min are shown in Figure 3C. For TC, in the presence of the FMN solution, two peaks of the mixed solution, 368 and 445 nm, were observed in the spectrum of the dark control, and the spectrum of the mixed solution with a maximal absorbance at 348 nm was inspected after 30 min of blue light irradiation. In our previous study, blue light-induced photolysis with FMN was found to generate ROS, and the spectrum absorbance of FMN was reduced drastically at 445 nm after blue light irradiation, which may have been due to the isoalloxazine ring photoreduction by electron migration from the ribityl side-chain of FMN [15]. TC was also unstable when irradiated by blue light, as seen in Figure 3B. In our previous study, the O_2_•^−^ was yielded from TC treated under blue light, and the molecular formula of the photolysis product was C_22_H_22_N_2_O_9_ [7]. As seen in Figure 3B,C, the spectrum absorbance of the TC treated without and with FMN was shifted from 368 to 348 nm after blue light irradiation for 30 min. It was uncertain whether the change of the photolytic product of TC occurred through the TC treated with or without FMN after 30 min of blue light irradiation in this section.

### 3.2. Detection of O_2_•^−^ via FMN or TC Photolysis

**Figure 4:**
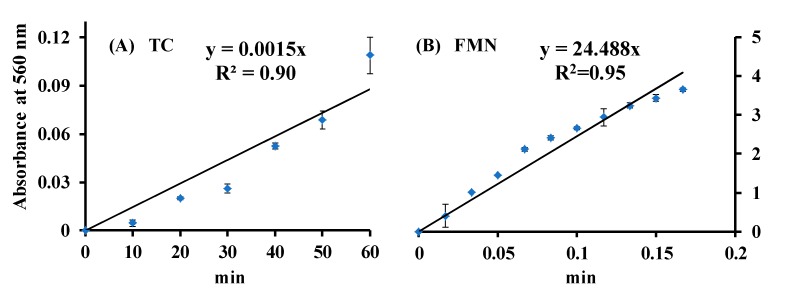
Effects of (**A**) TC and (**B**) FMN on nitro blue tetrazolium (NBT) reduction under blue light irradiation at 20 W/m^2^. The symbols (♦) in blue are the averaged absorbance of experimental data at different time points.

The generation of O_2_•^−^ from FMN and TC via blue light irradiation was studied by the NBT reduction method [7,15,27]. The reduction profiles in the FMN/NBT and TC/NBT systems under blue light irradiation for different time intervals are shown in Figure 4. In Figure 4, whether the blue light photoreduction of NBT in FMN or TC solution, an increasing time-dependent pattern was detected. It suggests that FMN or TC photolysis generates O_2_•^−^, which is involved in the photochemical system. The slopes of the NBT reaction curve of the ROS production for TC and FMN at the same conditions were 0.0015 and 24.488, respectively, as shown in Figure 4. 

### 3.3. Effects of TC and D-TCF on E. coli Viability 

**Figure 5:**
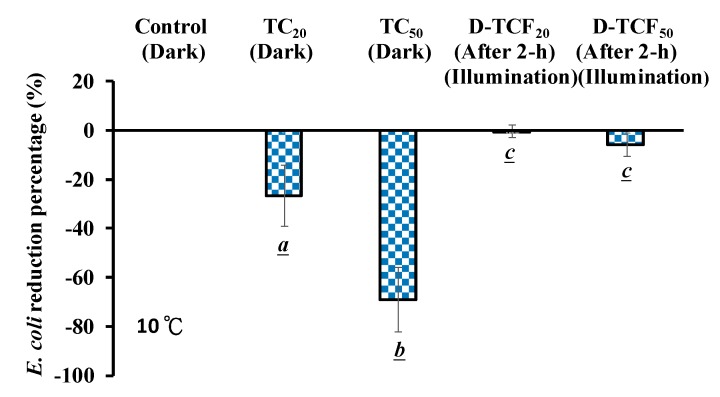
Effects of TC and degradation of TC (D-TCF) on *E. coli* viability at 10 ± 1 °C. Data are expressed as the mean ± SD, where *n* = 3. Statistically significant difference (*p* < 0.05) between an experimental group and the control is indicated by a letter below the each bar. Any two experimental groups that are significantly different between each other are labeled by different letters.

In this study, the microbial viability due to D-TCF (TC in 114 μg/mL of FMN solution at pH 7.8, treated under blue light at 20 W/m^2^, TCF treatment) was also explored. As shown in Figure 5, it seems that, for *E. coli*, the higher the concentration of the TC treatment, the higher the reduction percentage. The inactivation rates of 26.7% and 69.1% on *E. coli* were achieved via the 20 and 50 μg/mL TC treatments at 10 ± 1 °C, respectively, as shown in Figure 5. Under TCF treatment at 10 ± 1 °C for 2 h, the inactivation rates of 0.49% and 5.79% were achieved on *E. coli* via 20 and 50 μg/mL of D-TCF solution, respectively, but the inactivation rates of the two treatments were not significantly different (*p* > 0.05), as shown in Figure 5, implying that 20 and 50 μg/mL of TC treated with FMN solution, after blue light irradiation treatment at 10 ± 1 °C, undergo conformational changes. In our previous study, conformationally changed TC at 50 μg/mL enhanced the antibiotic ability of MDR *E. coli* upon blue light irradiation [7]. Comparing the changes of structure under the same condition, 50 μg/mL of TC treated with FMN solution after blue light irradiation at 25 °C was chosen in this study.

**Figure 6:**
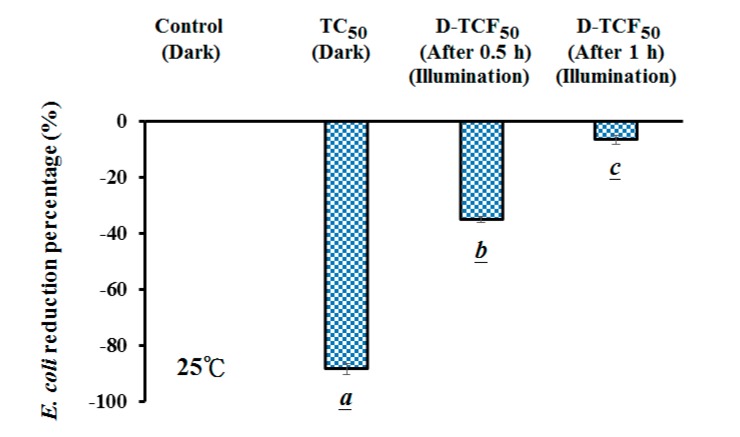
Effects of TC and D-TCF on *E. coli* viability at 25 ± 3 °C. Data are expressed as the mean ± SD, where *n* = 3. Statistically significant difference (*p* < 0.05) between an experimental group and the control is indicated by a letter below the bar. Any two experimental groups that are significantly different between each other are labeled by different letters.

The effects of D-TCF on the viability of *E. coli* at 25 ± 3 °C were also investigated. In Figure 6, the reduction percentage of *E. coli* was found to increase significantly with the TC treatment at room temperature. Under the TCF treatment at 25 ± 3 °C for 0.5 and 1 h, the inactivation rates of 35.2% and 6.8% on *E. coli* were achieved via 50 μg/mL of D-TCF solution, respectively, as shown in Figure 6.

### 3.4. Molecular Identification via LC-MS/MS Analyses

**Figure 7:**
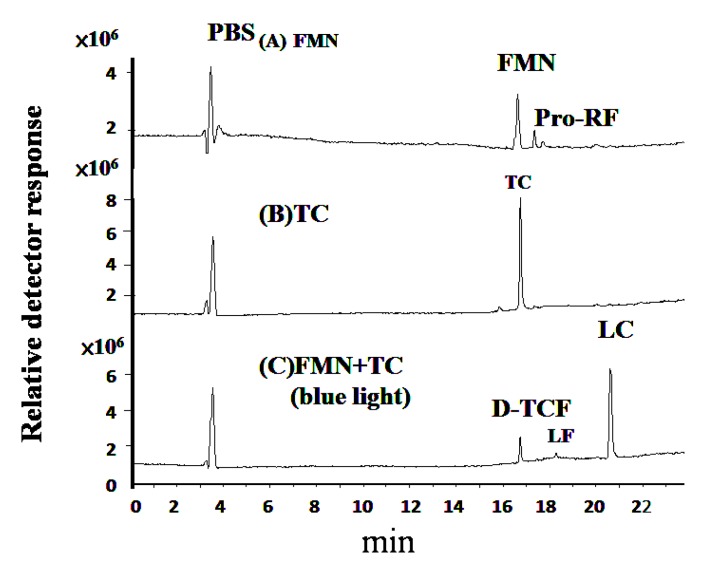
The chromatograms of the HPLC-MS analyses of (**A**) FMN (pH 7.8), (**B**) 50 μg/mL of TC solution, and (**C**) 50 μg/mL of TC in FMN solution after 20 W/m^2^ blue light irradiation at 25 ± 3 °C for 1 h. PBS: phosphate buffer solution. Notice that the Pro-RF in (**A**) coeluted with FMN is confirmed as riboflavin and described in the Results section.

The solutions of FMN and TC after blue light treatment were analyzed via LC-MS/MS. In Figure 7A, the total ion chromatogram of the PBS at pH 7.8 can be observed at 3.4 min. FMN is a derivative of riboflavin, obtained by the phosphorylation of the ribityl side-chain. In Figure 7A, the total ion chromatograms of FMN at pH 7.8, without photolysis processing, were observed at 16.63 min (FMN) and 17.376 min (Pro-RF). In Figure 7, the peak labeled as Pro-RF eluted at 17.376 min was identified as riboflavin by the major ion fragment at m/z 377.1, which is described in the Results section. As shown in Figure 7B, the TC at pH 7.8, without photolysis processing, was observed at 16.734 min. In Figure 7C, for the TC treated with FMN, the total ion chromatogram containing compounds eluted at 16.734 (D-TCF), 18.271 (LF), and 20.614 min (LC) after 20 W/m^2^ of blue light irradiation at 25 ± 3 °C for 1 h, as shown below.

**Figure 8:**
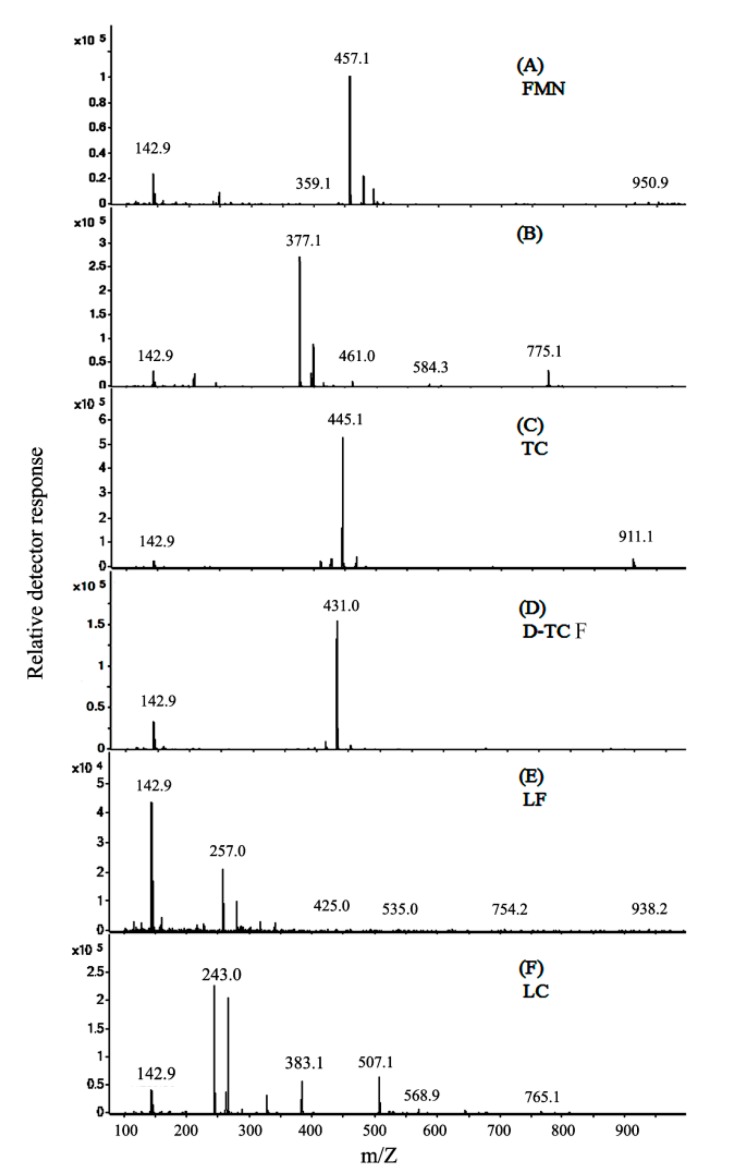
Spectra of (**A**) FMN, (**B**) riboflavin, (**C**) TC, (**D**) D-TCF, (**E**) lumiflavin, and (**F**) lumichrome from the TC treated with FMN under blue light irradiation for 1 h. The ionic spectral product of [M+H]^+^ is considered to be blue light-altered TC in solution (pH 7.8). The precursor ion of m/z 445 was selected, and the proposed fragments are shown in each spectrum.

In Figure 8, the electron ionization mass spectra, after the analyses of FMN and TC photolysis, are presented. The FMN is observed at 16.63 min, as shown in Figure 7A. The major ion fragment of FMN was at m/z 457.1, as shown in Figure 8A. The Pro-RF is observed at 17.376 min, as shown in Figure 7A. The compound of Pro-RF was confirmed by the major ion fragment at m/z 377.1, as shown in Figure 8B, and it thus could be identified as riboflavin. In Figure 7B, TC is observed at 16.734 min with the major ion fragment of m/z 445.1, as shown in Figure 8C. The D-TCF was noticed at 16.734 min via the TC treated with FMN photolysis, as shown in Figure 7C. The major ion fragment of the mass spectrum of D-TCF was at m/z 431.0, as shown in Figure 8D. The emerging peaks, LF and LC, were noticed at 18.271 and 20.614 min, respectively. Furthermore, the compounds of LF and LC were verified by their mass spectra, showing major ions of m/z 257.0 and 243.0, as seen in Figure 8E,F, respectively, and thus identified as lumiflavin and lumichrome.

## 4. Discussion 

FMN, a non-toxic micronutrient and a photosensitive compound, is essential for human bodily functions. A photosensitizer can be applied as an ingredient to catalyze the degradation of chemicals to produce reactive species, such as ROS, by irradiation. The current study showed that the antibacterial ability of TC can be decreased by blue light-induced ROS using FMN. The photolytic procedure of FMN can thus greatly reduce the antimicrobial capability of TC. Therefore, this method has the potential to be an environmentally sound method for wastewater treatment.

The molecular formula of TC is C_22_H_24_N_2_O_8_ (MW, 444.44 g/mol), and it is sensitive to blue light. For the TC in solution, the absorption of TC at 364 nm was considerably decreased, and O_2_•^−^ was generated via blue light irradiation, on the basis of this study. The effects caused by TC after blue light irradiation treatment at 20 W/m^2^ for 2 h (TC-B) on the survival rate of *E. coli* were significantly increased in our previous study [7]. Conformational changes of TC may influence its antibiotic effectiveness in the solution as well. Both TC and FMN are sensitive to blue light, so the photolytic productions of TC alone and TCF treatments were compared. The photolysis of TC alone under blue light irradiation did not cause much degradation of TC, with the main compounds being TC (MW, 444.44 g/mol) and the photochemical product of TC-B (MW, 458.44 g/mol), as shown in a previous study [7]. TC, in the presence of FMN, decomposed completely in the solution after blue light irradiation treatment, and the exact mass of the product (D-TCF) was 430.44 g/mol in this study.

**Figure 9:**
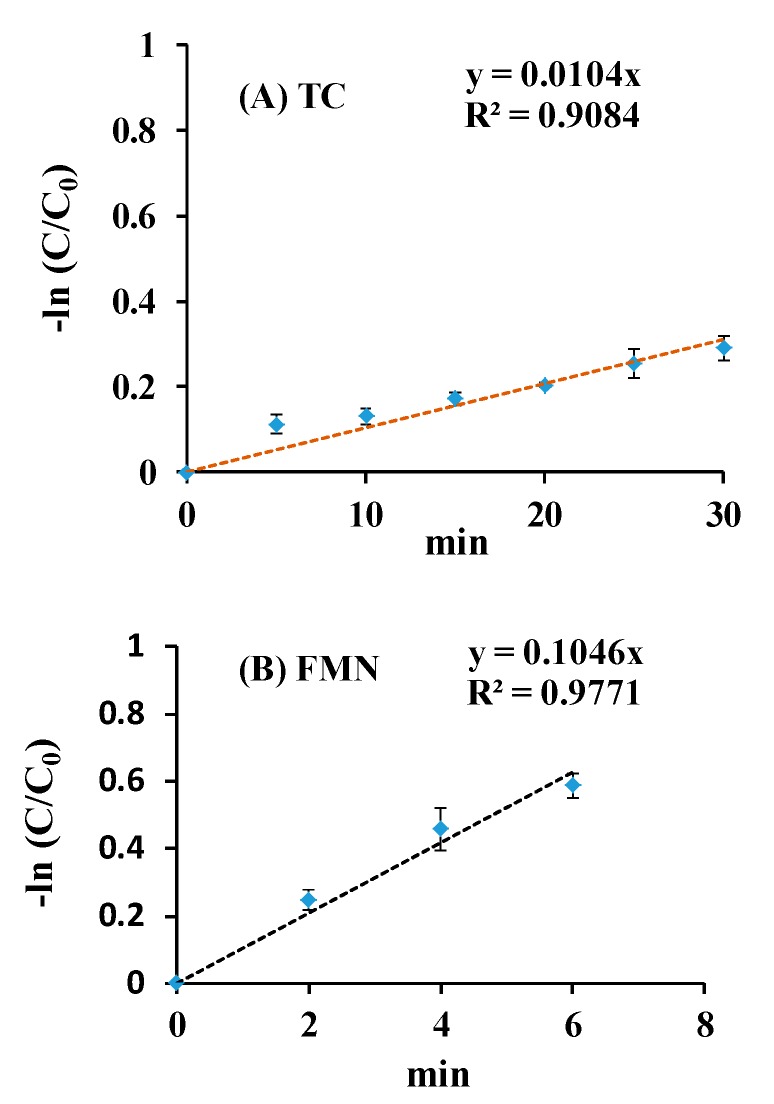
The k_appa._ of (**A**) TC and (**B**) FMN treated with blue light irradiation at 20 W/m^2^.

As shown in Figure 3B, the absorption at 364 nm was extensively decreased after 30 min of blue light irradiation, and the TC expression rate is assumed to follow a model that undergoes a pseudo-first-order kinetics (Equation (1)), as studied previously [7]. Equation (2) can be acquired, from *t* = 0 to *t* = t, by integrating Equation (1). Thus, the TC photolysis kinetics in PBS was imitated, according to a model of pseudo-first-order kinetics, shown below.

(1) $$-\frac{dC_{TC}}{dt}=k_{appa.}\times C_{TC},$$

(2) $$-\ln〔\frac{C_{TC}}{C_{0}}〕=k_{appa.}\times t.$$

where C_0_ and C*_TC_* are the primary concentrations of TC and the concentration of TC at the reaction time**,** respectively. The term k_appa._ is the rate constant of apparent decomposition. The peak at 445 nm was significantly reduced after 10 min of irradiation by blue light in the FMN solution, as shown in Figure 3A. k_appa._ from 50 μg/mL of TC or 50 μg/mL of FMN were used to compare the photolytic ability via blue light irradiation at 20 W/m^2^, as shown in Figure 9. As Figure 9 shows, the k**_appa_**. of TC or FMN, under blue light irradiation at 20 W/m^2^, were 0.0104 or 0.1046 (min^−1^), respectively. In addition, the kinetics of photolysis indicated that the photolysis efficiency of FMN was faster under blue light than that of TC. The formation of O_2_•^−^ was analyzed using the FMN/NBT and TC/NBT systems, as shown in Figure 4. The slope of the NBT reduction method of FMN was much steeper than that of TC in this study. That is, the level of ROS generation in FMN photolysis under blue light irradiation was much higher than what occurs in TC, and the better property of FMN photolysis, in terms of generating radical species, may be ascribed to its photosensitivity to blue light or perhaps any other photoradiation with sufficient frequency.

**Figure 10:**
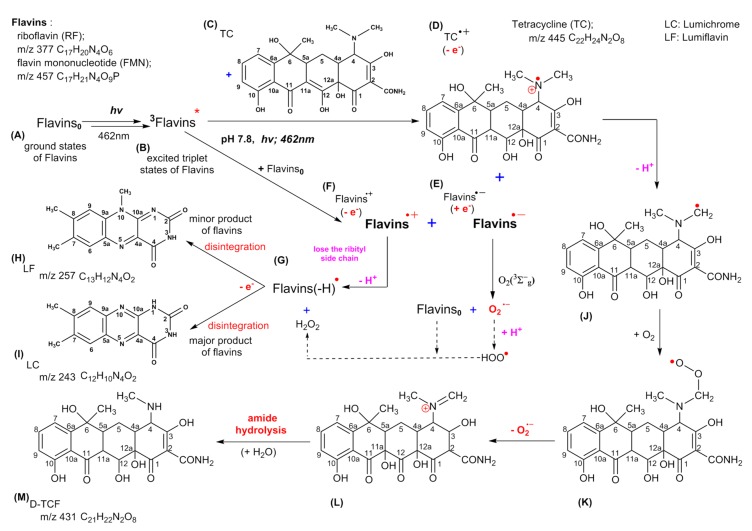
Proposed scheme for the photodegradation mechanisms of FMN/TC, where (**A**) is ground state of flavins; flavins_0_, (**B**) is excited triplet states of flavins; ^3^flavins*, (**C**) is tetracycline (TC), (**D**) is a semi-oxidized radical cation of tetracycline; TC•^+^, (**E**) is a semi-reduced radical anion of flavins; flavins•^−^, (**F**) is a semi-oxidized radical cation of flavins; flavins•^+^ (**G**) is the semi-oxidized radical cation of flavins by hydrogen atom abstraction; flavins(-H)^•^, (**H**) is a minor photo-degradation product of flavins; lumiflavin (LF) (**I**) is a major photo-degradation product of flavins; lumichrome (LC), (**J**–**L**) when a single electron transfer and hydrogen abstraction from the TC, exciting flavins under photolytic treatment, are able to follow a process with an iminium intermediate, (**M**) is an iminium intermediate that undergoes fast hydrolysis and become *N*-demethylation product (D-TCF).

According to the consequences of the current study, a scheme for the mechanism of FMN/TC photodegradation was proposed, as shown in Figure 10. The processes of the initial photochemical reactions of flavins are represented in Equation (3).

(3) $$\mathrm{flavins}\;+\;h\nu \;\to \;{}^{1}\mathrm{flavins}\to \;{}^{3}{\mathrm{flavins}}^{*}$$

where ^1^flavins^*^ and ^3^flavins^*^ are the excited singlet and triplet states of the flavins, respectively. The degradation mechanisms of these photoinduced flavins were likely initiated by a triplet−triplet annihilation or a triplet–ground state quenching process [25,31,32,33], the latter of which is depicted in this study. The TC directly oxidized by an electron abstraction through an electron transfer event from the TC ground state was started via flovin-sensitised irradiation [25]. TC and the ground state of flavins (flavins_0_) are the electron donors, which can offer an electron to ^3^flavins* and generate the semi-reduced (flavins•^−^) and semi-oxidized (TC•^+^, flavins•^+^) forms, respectively, as shown in Equations (4) and (5).

(4) $${}^{3}{\mathrm{flavins}}^{*}\;+\;\mathrm{TC}\;\to \;{\mathrm{flavins}}^{•-}\;+\;{\mathrm{TC}}^{•+},$$

(5) $${}^{3}{\mathrm{flavins}}^{*}\;+\;{\mathrm{flavins}}_{0}\;\to \;{\mathrm{flavins}}^{•-}\;+\;{\mathrm{flavins}}^{•+-}.$$

The photodegradation pathways of TC and flavins are achieved by flavin photosensitization. The TC and flavins were promoted to the electronically excited state, TC• and flavins•, via a photolytic process, and the flavins were reduced by losing an electron, as shown in Equations (6)–(14).

(6) $$\mathrm{TC}\;+\;h\nu \;\to \;{\mathrm{TC}}^{•},$$

(7) $${\mathrm{TC}}^{•}+\;\mathrm{flavins}\;\to \;{\mathrm{TC}}^{•+}\;+\;\mathrm{flavins}\left(e^{-}\right)\to \;{\mathrm{TC}}^{•+}\;+\;{\mathrm{flavins}}^{•-},$$

(8) $$\mathrm{TC}{}^{•+}\;\overset{-H^{+},\;O^{2},\;-O_{2^{•-}},H_{2}O}{\to }\;\mathrm{degraded}\;\mathrm{compounds},$$

(9) $$\mathrm{flavins}\;+\;h\nu \;\to \;{\mathrm{flavins}}^{•},$$

(10) $$\mathrm{flavins}{}^{•}\;+\;\mathrm{flavins}\;\to \;\mathrm{flavins}{}^{•+}\;+\;\mathrm{flavins}(e^{-})\;\to \;\mathrm{flavins}{}^{•+}\;+\;\mathrm{flavins}{}^{•-},$$

(11) $$\mathrm{flavins}{}^{•+}\;\overset{-H^{+}}{\to }\;\mathrm{flavins}(-H){}^{•}\overset{\;-e{}^{-}}{\to }\;\mathrm{degraded}\;\mathrm{compounds},$$

where TC•^+^ and flavins^•+^ are both radical and cation species.

Simultaneously, the interaction of flavins^•-^ with O_2_ (^3^Σg^−^) can generate the reactive superoxide ion, O_2_•^−^, followed by the hydroperoxyl radical, HOO•, which is generated by a reaction between O_2_•^−^ and a hydrogen atom and causes the degradation of flavins_0_.

(12) $$\mathrm{flavins}{}^{•-}\;+\;O_{2}({}^{3}\Sigma_{g^{-}})\;\to \;{\mathrm{flavins}}_{0}\;+\;O^{2}{}^{•-},$$

(13) $$O^{2}{}^{•-}\;+\;H^{+}\;\to \;\mathrm{HOO}{}^{•},$$

(14) $$\mathrm{HOO}{}^{•}+\;{\mathrm{flavins}}_{0}\;\to \;H_{2}O_{2}\;+\mathrm{flavins}\left(-H\right){}^{•}\;\overset{\;-e{}^{-}}{\to }\;\mathrm{degraded}\;\mathrm{compounds}.$$

where flavins^•−^ is a radical anion.

As depicted in Figure 10, flavins treated with blue light irradiation can reach an electronically excited state (flavins*), whereas the abstraction in the selective C–H bond of the *N*-methyl group in TC can form an aminium radical cation via an initial single electron transfer, as shown in Figure 10D [34]. A further deprotonation (-H^+^) of α-C leads to the formation of a carbon-centered radical. The carbon-centered radical reacts rapidly with dissolved oxygen to generate a peroxyl radical, perhaps followed by the loss of a superoxide radical (O_2_•^−^) to yield an iminium cation. It is well known that imine, due to its chemical instability in aqueous solution, undergoes fast hydrolysis, as the aforementioned *N*-demethylation product, D-TCF (m/z 431), is formed, as shown in Figure 10M. In short, a single electron transfer and hydrogen abstraction from the TC, exciting flavins under photolytic treatment, are able to follow a process with an iminium intermediate, which later underwent hydration and oxidation to become additional intermediates, as shown in Figure 10J–L, and generated the demethylated product [35]. 

Huvaere et al. (2010) [36] reported that the electron transfer from flavins_0_ to the oxidizing ^3^flavins* can generate a reduced flavin radical (flavins^•**−**^), which can be readily oxidized by residual oxygen to regenerate the incipient flavins_0_ and the concomitant formation of superoxide (O_2_•^−^), as shown in Figure 10E. The highly reactive flavin radical cation (flavins^•**+**^) was generated simultaneously under the photolytical process with a self-quenching (oxidized flavin) reaction, as shown in Figure 10F.

The flavins^•**−**^ were readily oxidized to generate the incipient flavins_0_ and O_2_•^−^. The reactive hydroperoxyl radical (HOO•) could be formed via the process in which a proton is added to O_2_•^−^, leading to the degradation of flavins, as shown in Figure 10G. The flavins(-H)^•^ could also be produced via hydrogen atom abstraction from the flavins^•+^. In that case, the flavins(-H)^•^ would release an electron, which would cause the degradation of flavins, as shown in Figure 10G [32]. In photodegradation of flavins, the flavin solution disintegrates into a series of products via the light-induced autoreaction with the most characteristic molecular ions ([M+H]^+^) thus formed, that is, m/z 257 and m/z 243, corresponding to the formation of lumichrome (LC) and lumiflavin (LF), as shown in Figure 10H,I, respectively [36].

As shown in Figure 5 and Figure 6, the inactivation rates of 69.1% and 90.1% for *E. coli* were achieved via 10 and 25 °C for 50 μg/mL of TC treatments, respectively. It seems that for *E. coli*, the higher the temperature treatment of TC, the higher the reduction percentage. As shown in Figure 5, a 94.3% viability of *E. coli* was retained in the TCF treatment with 20 W/m^2^ blue light irradiation at 10 °C for 2 h (14.4 J/cm^2^, high-energy dose). The antibiotic ability of TC for *E. coli* was noticed as increasing in a temperature-dependent manner in this study. However, for the TCF treatment with 20 W/m^2^ of blue light irradiation at 25 °C for 1 h (7.2 J/cm^2^, low-energy dose), a 93.2% viability of *E. coli* was shown in this study. At a low-energy dosage, the photosensitizer might use FMN treated at a high temperature to increase *E. coli* viability. This suggests that the photodegradation of TC in FMN solution may also reduce its antibiotic property in relation to microorganisms at room temperature.

It has been reported that, for the structurally-related activity of TC, each of the rings in the linearly fused tetracyclic nucleus must be six-membered for the molecules to maintain their antibacterial activity [1]. The major ion fragment of TC was found to be m/z 445.1. However, after the TCF treatments at 25 °C for 0.5 and 1 h, TC was found to be 99.4% and 100% decomposed, respectively, in this study. The major ion fragment of the mass spectrum of D-TCF was m/z 431.0, as observed via the TCF treatment in this study. However, after the TCF treatments at 25 °C for 0.5 and 1 h, the viabilities rates of 64.8% and 93.2% for *E. coli* were achieved via 50 μg/mL of D-TCF solution. It was suggested that TCF treatment reduces the antibiotic property of TC in an energy-dependent manner, and blue light irradiation and the D-TCF from the TCF treatment had less antibacterial activity in relation to *E. coli*. 

TC and FMN are both sensitive to blue light irradiation, with O_2_•^−^ being produced via the photolytic process. It has been reported that demeclocycline is a semisynthetic tetracycline that is photosensitive to violet light (415 nm). ROS generated from demeclocycline photolysis under violet light irradiation can inactivate methicillin-resistant *S. aureus* (MRSA) and *E. coli* bacteria [37]. FMN is also sensitive to violet light, with a higher efficiency in the inactivation of *S. aureus* via the decomposition of FMN by photolysis, as compared to blue light irradiation at the same radiant intensity [27]. It is therefore of interest in the study of the conformational changes of TC and its antibiotic behavior upon the FMN photolytic process under violet irradiation. 

The wavelength of the maximum emission of blue light applied in this study was 462 nm. It is well known that the white LED spectrum is a combination of different color lights. The efficiency of FMN photolysis by white LED light was found to be about 75%, which is similar to that of FMN photolysis by blue LED light at the same intensity [15]. As an energy-efficient light source, white LED lamps can be applied to replace a blue LED light source for TC degradation during FMN photolysis treatment, though its efficiency is lower [10]. Blue light irradiation at 20 W/m^2^ for 2 h had almost the same efficiency as solar radiation by sunshine for 30 min in terms of crystal violet degradation during riboflavin photolysis, occurring together with ROS generation [15]. ROS generated from the NBT reduction method were relatively lower in riboflavin than they were in FMN at an equivalent illumination level. When the sun is shining brightly, sunlight could be the primary source of radiation for TC degradation under the FMN photolytic process.

## 5. Conclusions

This study established that FMN photolysis with blue light (λ = 462 nm) irradiation degrades TC by ROS generation. For TC in the presence of FMN in a phosphate solution irradiated by blue light, the structural change of TC occurred via the pathways involving ROS. TC degradation caused by FMN photolysis protocols can highly decrease the antimicrobial ability of TC and conceivably reduce its influence on ecosystems containing environmental microflora. Therefore, FMN photolysis treatment under blue light irradiation is an easy and a secure means to decrease the antimicrobial ability of TC in solution and perhaps other types of antibiotic compounds.
